# A Novel Full-length IgG Recombinant Antibody Highly Specific to Clothianidin and Its Application in Immunochromatographic Assay

**DOI:** 10.3390/bios12040233

**Published:** 2022-04-11

**Authors:** Yunyun Chang, Yang Chen, Shasha Jiao, Xinying Lu, Yihua Fang, Yihua Liu, Ying Zhao, Xiuping Zhan, Guonian Zhu, Yirong Guo

**Affiliations:** 1 Institute of Pesticide and Environmental Toxicology, Key Laboratory of Biology of Crop Pathogens and Insects of Zhejiang Province, Ministry of Agriculture Key Laboratory of Molecular Biology of Crop Pathogens and Insects, Zhejiang University, Hangzhou 310058, China; 21716193@zju.edu.cn (Y.C.); chengyang96@zju.edu.cn (Y.C.); 21616183@zju.edu.cn (S.J.); 12016122@zju.edu.cn (X.L.); 0915059@zju.edu.cn (Y.F.); zhaoying88@zju.edu.cn (Y.Z.); zhugn@zju.edu.cn (G.Z.); 2 Research Institute of Subtropical Forestry, Chinese Academy of Forestry, Hangzhou 311400, China; 3 Shanghai Agricultural Technology Extension Service Center, Shanghai 201103, China; zxp19830124@aliyun.com

**Keywords:** clothianidin, heterologous *ic*-*ELISAs*, recombinant full-length IgG, non-competitive surface plasmon resonance assay, gold immunochromatographic assay

## Abstract

The toxicity of clothianidin to non-target organisms has gradually attracted world-wide attention. It is essential to develop reliable methods for the on-site detection of clothianidin residue. In this study, analogue-based heterologous *ic*-*ELISAs* were designed to rapidly screen desirable hybridomas, which could be used for the construction of recombinant antibodies (RAbs) against clothianidin. Based on the antibody variable region genes, two full-length IgG RAbs (1F7-RAb and 5C3-RAb) were produced by the mammalian cell expression system. The performance of the two RAbs was characterized and compared by heterologous *ic*-*ELISAs* and non-competitive surface plasmon resonance (SPR) assays. Using heterologous *ic*-*ELISAs*, the 1F7-RAb exhibited highly specific and sensitive recognition to clothianidin with an IC_50_ of 4.62 μg/L, whereas the 5C3-RAb could bind to both clothianidin and dinotefuran. The results of the non-competitive SPR assay further verified that the 1F7-RAb had a higher specificity and affinity to clothianidin than the 5C3-RAb. Finally, a gold immunochromatographic assay based on the novel antibody, 1F7-RAb, was developed for rapid detection of clothianidin with high sensitivity (visual detection limit of 2.5 μg/L), specificity, and good reproducibility, which can be used as an effective supervision tool for clothianidin residue in agricultural and environmental samples.

## 1. Introduction

As an alternative to instrumental detection methods, immunoassays show great potential for on-site screening tests in the fields of environmental monitoring and food safety control [1,2] because they are rapid, sensitive, simple, and cost-effective. The excellent properties of immunoassays are dominantly determined by the involved antibodies. Thus, an investigation of antibodies against target analytes is the key to immunoassay development. Traditional antibodies including monoclonal antibodies (McAbs) and polyclonal antibodies (PcAbs) are common reagents for immunoassay development. However, they are facing some challenges. For instance, animal-dependent PcAbs are inclined to batch differences in functionality and relatively poor specificity, whereas the preparation of McAbs is overly dependent on the performance of hybridoma cells, which sometimes appear with variable region gene diversity, mutation, or even loss resulting in unstable antibodies [3,4]. If any of these problems occur, there is no doubt that all processes need to be restarted to produce new antibodies, which to some extent, hinders the application of traditional antibodies in immunoassay development.

With the advancement of genetic engineering techniques, recombinant antibodies (RAbs) provide another promising alternative to traditional antibodies for immunoassay development, avoiding unstable factors [5]. The antibody genes can be derived from hybridomas or B lymphocytes for the construction of RAbs, and the former is more convenient. Once the gene sequences are successfully obtained, immortalized RAbs can be produced by in vitro expression systems with improved reproducibility, which is beneficial to the establishment of stable immunoassays with standardized reagents. RAbs have been increasingly studied and used in immunoassays for pesticide residues [6,7]. However, there are rare reports on the RAbs’ application to gold-labeled immunochromatographic assays (GICA), the most popular rapid tests [8,9,10]. This may be due to the fact that most of the previous RAbs against pesticides are fragment RAbs, such as single-chain variable fragments (ScFv), with a weaker affinity than parental McAbs, PcAbs, or full-length IgG antibodies. Recently, full-length RAbs against small-molecule hazards have been studied [11,12], which can be an alternative for fragment RAbs as the key reagent to develop standardized rapid tests.

Clothianidin is one of the typical neonicotinoid insecticides widely applied to control thrips, leafhoppers, and other agricultural pests. Also known as the main metabolite of thiamethoxam [13] (another neonicotinoid extensively used as a seed treatment), clothianidin has accumulated in the environment, including in agricultural products [14], rivers [15], soil [16], and even the air [17], posing a potential threat to ecological and human health. Previous studies have revealed that clothianidin has adverse effects on non-target organisms such as honey bees [18,19], soil invertebrates [20], birds, and butterflies [21]. Thus, in recent years, many countries have restricted or banned the use of clothianidin, a controversial insecticide. For instance, since 2018 the European Union (EU) has banned the use of clothianidin in outdoor farming because of its toxicity to honey bees (the most effective pollinators). Currently, immunoassays for clothianidin have been reported, which are based on traditional antibodies with some unstable factors. Furthermore, these antibodies have a significant cross-reaction with dinotefuran, a new neonicotinoid insecticide that is increasingly used. Consequently, the developed immunoassays based on these antibodies cannot distinguish clothianidin and dinotefuran accurately [22,23,24,25], easily resulting in a false-positive determination. Hence, it is urgent to develop novel anti-clothianidin antibodies with high specificity, high affinity, and stable performance for immunoassay development to regularly monitor clothianidin residue in an ecological environment.

In this study, we firstly screened out two hybridoma lines that secreted high-affinity and specific antibodies against clothianidin by employing an analogue-based heterologous strategy. Based on the antibodies’ variable regions (VR) of hybridomas, the corresponding full-length IgG RAbs (1F7-RAb and 5C3-RAb) were constructed by a mammalian cells expression system with heavy and light chains from two separate vectors. The antibody performances were characterized and compared by an indirect-competitive enzyme-linked immunosorbent assay (*ic*-*ELISA*) and a non-competitive surface plasmon resonance (SPR) assay. Further, 1F7-RAb with higher specificity and affinity was used to develop a reliable GICA for the sensitive and accurate detection of clothianidin residues in environmental and agricultural samples.

## 2. Materials and Methods

### 2.1. Reagents and Materials

Standards of clothianidin (99.5%) and other neonicotinoids pesticides were purchased from Dr. Ehrenstorfer GmbH (Augsburg, Germany). Horseradish peroxidase (HRP)-goat anti-mouse, ovalbumin (OVA; MW 45,000), and bovine serum albumin (BSA; MW 67,000) were purchased from Sigma-Aldrich (Madrid, Spain). RNAiso Rea-gent, SMARTer TM RACE cDNA amplification kit MiniBEST Agarose gel DNA extraction kit, and In-Fusion HD cloning plus kit were bought from Takara (Tokyo, Japan). Trans1-T1 phage resistant chemically competent cell and pEASY^®^-blunt zero cloning kit were purchased from TransGen Biotech (Beijing, China). Restriction endonucleases including HindIII and EcoRI were purchased from New England Biolabs (Ipswich, MA, USA). Protein A Agarose Resin 4FF was purchased from Yeasen Biotechnology (Shanghai, China). QIAGEN Plasmid Mini Kit was obtained from QIAGEN biotechnology (Duesseldorf, Germany). Expression vectors, pCDNA3.4-Mouse-IgG1-CH and pCDNA3.4-Mouse-Kappa-CL were purchased from Biointron (Taizhou, China).

### 2.2. Hybridoma Screening

The haptens of clothianidin, imidaclothiz, and thiacloprid were prepared according to previous methods [22,26,27]. Clothianidin hapten conjugated with bovine serum albumin (CLO-H-BSA) was produced using carbodiimide method to serve as an immunogen, whereas the haptens of clothianidin, imidaclothiz, and thiacloprid conjugated with ovalbumin (CLO-H-OVA, IMI-H-OVA, and THC-H-OVA) were prepared using mixed anhydride method to serve as homologous and heterologous competitive antigens. Based on hybridoma technique, hybridoma cells were obtained by fusing mouse spleen cells with mouse myeloma cells. The hybridomas secreting specific antibodies to clothianidin were selected via homologous and heterologous *ic*-*ELISAs*, described in detail in Supplementary Materials. After three rounds of limiting dilution, the high-performance cell lines were used to produce mouse ascitic McAbs and chosen for the following study.

### 2.3. Sequencing and Analysis of Antibody VR Genes

Total RNAs of hybridoma cells were obtained via RNAiso Reagent. Rapid amplification of cDNA ends (RACE) kit was used to amplify the 5′end of cDNA of hybridoma cells. Polymerase chain reaction (PCR) program was then done to amplify VR of heavy chain (VH) and light chain (VL), with subtype-specific primers (VH-Primer1: CTCAATTTTCTTGTCCACCTTGGT; VL-Primer2: CTCATTCCTGTTGAA-GCTCTTGACAATGGG; VL-Primer3: CTCATTCCTGTTGAAGCTCTTGACGAC-GGG). The details were as follows: 25 cycles of 95 °C for 45 s, 68 °C for 45 s, and 72 °C for 3 min. The PCR products were identified by gel electrophoresis and subsequently purified with the gel extraction kit. After that, purified gene fragments were cloned into pEASY-Blunt vector and transformed into chemically competent E. coli T1 cells for sequencing.

The NCBI database was applied to analyze the data of antibodies’ VR genes and the classification of complementarity determining regions (CDRs), and framework regions (FRs) were conducted by Kabat rule.

### 2.4. Cloning Antibody Genes into Full-Length RAbs

#### 2.4.1. Construction of Expression Vectors

Both pCDNA3.4-Mouse-IgG1-CH and pCDNA3.4-Mouse-Kappa-CL were treated with double restriction endonuclease digestion (HindIII/EcoRI), and then respectively inserted with VH and VL using homologous recombination technology. Subsequently, recombinant plasmids were cloned into *E. coli* T1 and sequenced. Then, the recombinant plasmids with correct antibody gene sequences were extracted using QIAGEN Plasmid Plus Midi Kit.

#### 2.4.2. Production of Full-Length RAbs

A mammalian HEK293 (F) cell expression system was used to produce the full-length RAbs to functionally verify VR genes of antibodies according to our reported protocol [11], followed by the purification with protein A affinity chromatography.

### 2.5. Functional Analyses of Full-Length RAbs

#### 2.5.1. Heterologous *ic*-*ELISAs*

Checkerboard method was used to choose the optimization of antibody-antigen working concentration. The standard curve and regression equation were fitted by four-parameter equation via Origin 2017.

#### 2.5.2. Non-Competitive SPR

Non-competitive SPR was used to measure the binding properties of antibodies against clothianidin and other neonicotinoid insecticides using Biacore T200 instrument (GE Healthcare, Chicago, IL, USA). Each antibody was individually immobilized onto the different channels of the Series S CM7 sensor chip surface. The selectivity and kinetics affinity assays were carried out according to our previous studies [28,29] and the details and methods are described in the Supplementary Materials. After that, the dissociation equilibrium constant (KD), dissociation rate (kd), and association rate (ka) were obtained.

### 2.6. Development of Full-Length RAb-Based GICA for Clothianidin Detection

GICA for clothianidin was developed according to our previous study [30]. The experimental steps are shown in the Supplementary Materials. The most suitable pH (7.0) value and the optimal amount of antibody (70 μg/mL) for labeling were determined prior to formal experiments. The IMI-H-OVA at 0.15 mg/mL and goat anti-mouse IgG at 0.09 mg/mL were immobilized on the strip zones of test line and control line, respectively.

#### 2.6.1. Selectivity and Sensitivity Experiment

The selectivity of GICA was evaluated by detecting analogues (1000 μg/L) of clothianidin and the sensitivity was tested by a series of concentrations (0, 0.32, 0.63, 1.25, 2.5, 5, 10, 20, and 40 μg/L) of clothianidin standard in the optimal working solution. After 10 min, the results were judged with the naked eye according to the judgment standards (Figure S2). In order to investigate the influence of different batches of full-length RAbs on the reproducibility of the immunoassays, four batches of GICA strips were established based on four batches of full-length RAbs.

#### 2.6.2. Sample Matrix Effect Analysis

To reduce the matrix effects, environmental samples and homogenized agricultural samples (clothianidin-free) were added with 0.01 M PBS buffer, set to a different dilution multiple and then adjusted to pH 7 to prepare diluted samples. The clothianidin standard solutions were then prepared by diluted samples and applied for the GICA test.

#### 2.6.3. Analysis of the Spiked Samples

To determine the accuracy of the GICA, clothianidin standard solutions at different final concentrations were spiked into the water (5, 10, 20 ng/mL), soil (10, 20, and 40 ng/g), tomatoes (15, 30, and 60 ng/g), and oranges (15, 30, and 60 ng/g). The fortified samples were stored overnight and extracted with 0.01 M PBS buffer with 10% methanol. After ultrasonication for 3 min, these samples were then violently shaken for 3 min. The extracted solutions were then centrifuged at 4000 *g* for 10 min. The supernatant solutions were diluted for suitable dilution time and adjusted to pH 7 for GICA analysis.

## 3. Results and Discussion

### 3.1. Hybridoma Screening

**Figure 1:**
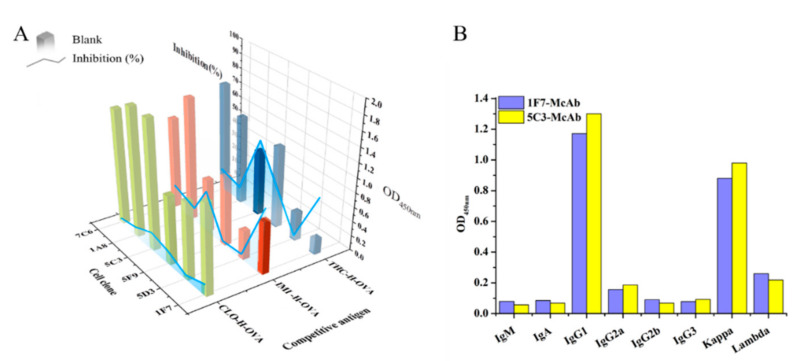
Characterization of antibodies from hybridoma supernatants. Screening hybridoma using *ic*-*ELISAs* with different competitive antigens. The concentration of competitive antigen was 5 μg/mL. The inhibition was tested with 50 μg/L of clothianidin (**A**). The isotypes determination of two antibodies (**B**).

After cell fusion, homologous *ic*-*ELISAs* were first carried out to screen hybridomas with CLO-H-OVA (5 μg/mL) as a competitive antigen. Six potential antibodies from hybridoma culture supernatants exhibited a strong positive response to CLO-H-OVA, with the optical density values at 450 nm (OD_450nm_) above 0.95 (Figure 1A). However, the free clothianidin (50 μg/L) could not inhibit the binding of CLO-H-OVA to antibodies with inhibition of less than 11%. Thus, at the primary stage, we could hardly distinguish the hybridoma clones secreting high-affinity antibodies specific to free clothianidin.

In our previous study [31], we found that the sensitivity of immunoassays could be improved by using analogue-based heterologous competitive antigens. The haptens of the analyte and its analogue shared the same linker for conjugation with the carrier protein so that the same chemical reaction was easily conducted for hapten synthesis without the additional design of a new heterologous linker that was always considered for the sensitivity improvement in other studies [32,33,34]. In this study, we attempted to design and conduct analogue-based heterologous *ic*-*ELISAs* to screen out the hybridomas of interest. By analyzing the chemical structures of eight common neonicotinoid pesticides (Table 1), imidaclothiz with a chlorinated thiazole ring and the nitroguanidine group have high similarity in chemical structures to clothianidin, whereas thiacloprid has low similarity to clothianidin. Thus, we finally selected the haptens of the two analogues (Figure S1) as representatives to prepare the heterologous competitive antigens (IMI-H-OVA and THC-H-OVA).

In heterologous *ic*-*ELISAs* with the same experimental conditions as homologous *ic*-*ELISAs*, the binding of the antibody from the 1F7-cell clone to IMI-H-OVA was inhibited 74.63% by free clothianidin, and free clothianidin inhibited the binding of the antibody of the 5C3-cell clone to IMI-H-OVA and THC-H-OVA with 51.9% and 53.72%, respectively (Figure 1A). The results show that the heterologous *ic*-*ELISAs* based on the structural-analogue competitive antigen were an effective strategy to identify high-affinity antibodies against free small-molecule analytes in hybridoma screening by minimizing the binding of the antibodies to the competitive antigen.

For further study, IMI-H-OVA was chosen as the heterologous competitive antigen for antibodies derived from the 1F7 clone, and THC-H-OVA causing the greatest inhibition effect was chosen for antibodies derived from the 5C3 clone. The cell clones 1F7 and 5C3 were used to prepare ascitic McAbs, namely 1F7-McAb and 5C3-McAb which were determined as both IgG1 heavy chain and κ light chain (Figure 1B).

### 3.2. Sequencing of VR Genes and Expression of Full-Length IG RAbs

**Figure 2:**
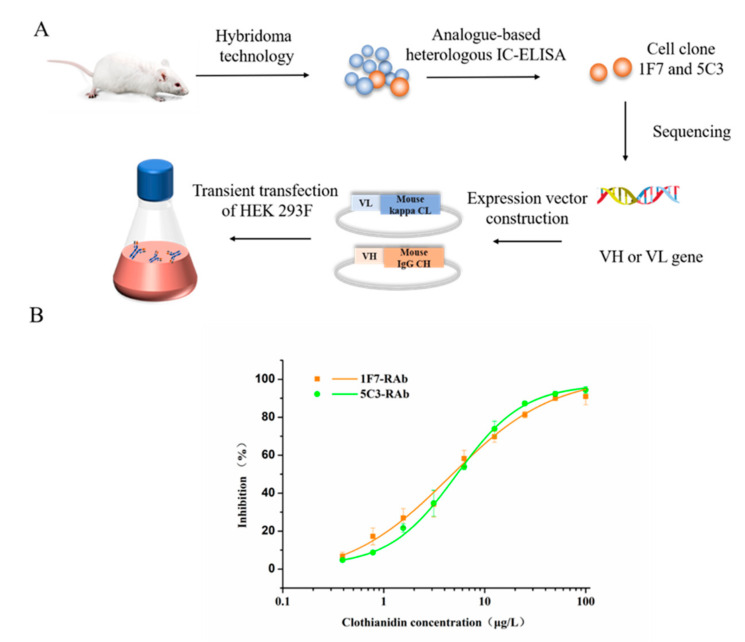
Cloning variable region genes of antibody from hybridoma for developing full-length IgG RAbs. Scheme of the production of full-length IgG RAbs (**A**). The inhibition curves of clothianidin were measured by antibodies based on heterologous *ic*-*ELISAs* (**B**). IMI-H-OVA-based heterologous *ic*-*ELISAs* for 1F7-RAb (1.25 μg/mL of IMI-H-OVA). THC-H-OVA-based heterologous *ic*-*ELISAs* for 5C3-RAb (5 μg/mL of THC-H-OVA).

As shown in Figure 2A, the total RNAs of the hybridoma cells 1F7 and 5C3 were successfully isolated and reversely translated into cDNA fragments. The VH and VL gene sequences of the antibodies were amplified by 5′RACE PCR (Figure S2). The gene sequences were translated into amino acid sequences and sequence alignment is shown in Figure S3. The results showed that the amino acid sequences of the light chains of the two antibodies had 92.9% identity and 99.1% similarity, whereas the heavy chains only had 43% identity but 82.6% similarity. The differences in the VH sequences must lead to different selectivity and affinity between the two antibodies toward the analytes.

To produce RAbs the correct expression plasmids were transfected into HEK293 (F) for transient expression, and the supernatant was collected and purified. The purified full-length IgG RAbs, namely 1F7-RAb and 5C3-RAb, were identified by gel electrophoresis with around 25 kD of light chain and 50 kD of heavy chain as expected (Figure S4). After purification of the cell supernatant, the production yields of 1F7-RAb and 5C3-RAb were 1.5 and 16 mg/L, respectively. This suggests that mammalian cells such as HEK293 (F) have a sequence preference for expressing the same type of protein, leading to different levels of expression.

### 3.3. Heterologous ic-ELISAs for Functional Characterization of Full-Length RAbs

After the optimization of the antibody–antigen working concentration, the standard curves of clothianidin were individually established by heterologous *ic*-*ELISAs*. As shown in Figure 2B, for the IMI-H-OVA-based heterologous *ic-ELISA* (1.25 μg/mL of IMI-H-OVA), the IC_50_ value of the 1F7-RAb for clothianidin standards was 4.62 μg/L. For the THC-H-OVA-based heterologous *ic*-*ELISA* (5 μg/mL of THC-H-OVA), the IC_50_ value of the 5C3-RAb was 5.2 μg/L. The sensitivity (IC_50_) of both antibodies for clothianidin was similar to or better than previously reported anti-clothianidin antibodies [22,23,35].

The other seven neonicotinoid insecticides were tested for cross-reactivity. As listed in Table 1, the 5C3-RAb showed a high cross-reactivity to dinotefuran (56%), similar to previously reported anti-clothianidin antibodies, whereas the 1F7-RAb showed no obvious cross-reaction to dinotefuran and could be considered the most specific antibody against clothianidin, which was beneficial to the establishment of an accurate immunoassay for clothianidin. Although the hapten for immunization was chemically identical, the 1F7-RAb produced in this study had a higher specificity than the 5C3-RAb or the other antibodies reported in previous studies [22,23,35], which showed the great diversity of antibodies from different organisms. Also, it is important to use different strategies to screen out antibodies with different performances.

### 3.4. Non-Competitive SPR Assay for Functional Characterization of Full-Length RAbs

**Figure 3:**
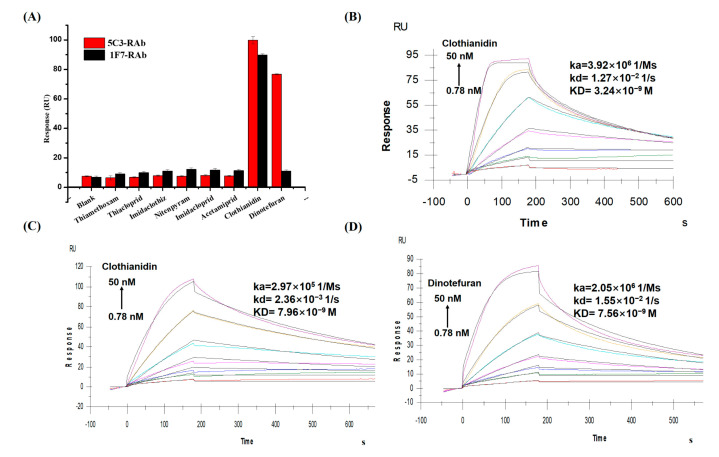
Full -length RAbs with analytes measured by non-competitive SPR. Binding selectivity of antibodies towards 8 neonicotinoids at the same concentration of 50 nM (**A**). Kinetic affinity tests of 1F7-RAb with clothianidin (**B**); 5C3-RAb with clothianidin (**C**); 5C3-RAb with dinotefuran (**D**). ka: association rate; kd: dissociation rate; KD: dissociation equilibrium constant.

Ideally, SPR-based direct analysis was good for studying the interaction between the antibody and the analyte itself. For the binding selectivity assay, as shown in Figure 3A, the 1F7-RAb only showed a high signal towards clothianidin, whereas the 5C3-RAb showed a high signal towards both clothianidin and dinotefuran, which was consistent with the results of the *ic*-*ELISAs*. For the kinetic evaluation, the 1F7-RAb exhibited higher affinity (KD = 3.24 × 10^−9^ M; Figure 3B) towards clothianidin than the 5C3-RAb (KD = 7.96 × 10^−9^ M; Figure 3C). In addition, 5C3-RAb showed a similar affinity to both clothianidin and dinotefuran (KD = 7.56 × 10^−9^ M; Figure 3D). By comparison of the kinetic parameters (k_a_, k_d_), the immunocomplexes of clothianidin and 5C3-RAb dissociated slower than that of dinotefuran and 5C3-RAb, indicating that the binding of 5C3-RAb to clothianidin was more stable than that to dinotefuran, which could be responsible for the IC_50_ value of 5C3-RAb to clothianidin being lower than to dinotefuran in the *ic*-*ELISAs*.

Compared to other previous studies [36,37,38], the antibodies’ VRs genes are always in vitro expressed in fragment RAbs formats, such as ScFv and Fab, for activity verification of the VH and VL sequences and RAbs-based immunoassay development. However, some fragment RAbs are not as specific and sensitive as their parental McAbs or full-length RAbs [12,39]. In this study, the 1F7-RAb and the 5C3-RAb exhibited very similar affinities and specificities to their parental McAbs, as measured by both *ic*-*ELISAs* (Table 1) and non-competitive SPR (Figure 3 and Figure S5). Thus, the full-length RAbs with a stable reproducibility, could be novel reagents as alternatives to hybridoma-dependent McAbs or fragment RAbs for developing immunoassays. Furthermore, the characterization results confirmed that the correct VR genes of antibodies from hybridomas had been successfully cloned and sequenced, providing a credible basis to study the recognition mechanism of antibodies, which will guide further antibody affinity maturation in vitro.

### 3.5. Application of a Highly Specific RAb in a GICA for Clothianidin Detection

#### 3.5.1. Specificity and Sensitivity of GICA

**Figure 4:**
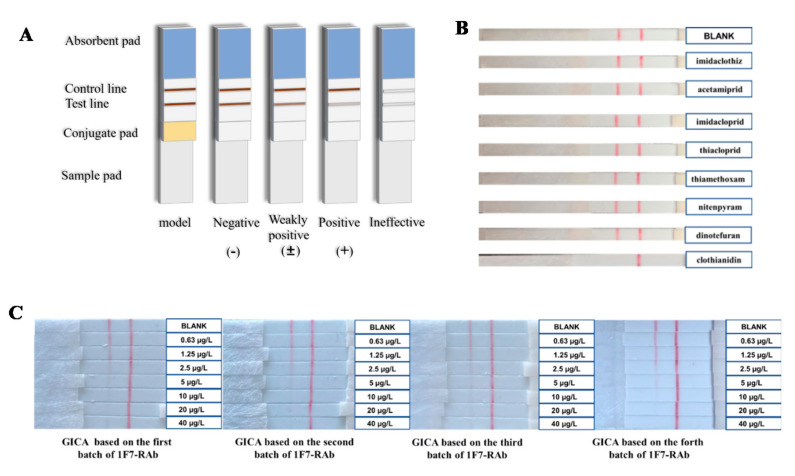
Tests using GICA. (**A**) Schematic diagram and the results of GICA. (**B**) Cross-reaction test of 1F7-RAb-based GICA to clothianidin and 7 analogues at 1000 μg/L. (**C**) Four batches of GICA tests for the standard solutions of clothianidin at 0, 0.63, 1.25, 2.5 5, 10, 20, and 40 μg/L.

Figure 4A shows the results of the GICA. As shown in Figure 4B, seven analogues at 1000 μg/L did not cause the disappearance of the T line, whereas clothianidin at the same concentration caused the complete disappearance of the T line. Thus, these cross-reaction results demonstrated that the GICA was highly specific to clothianidin, which is consistent with the above results of the SPR and the *ELISA*.

The lowest concentration of clothianidin that caused the markedly weaker color of the T line was regarded as the visual assay sensitivity. As shown in Figure 4C, when the concentration of clothianidin increased, the color of the T line became weaker and faded further. Compared to the blank control and the C-line, when clothianidin was 2.5 μg/L, the T line had an obvious color fade, and when clothianidin was more than 10 μg/L, the T line was invisible. The results indicated that the visual detection limit (LOD) of clothianidin by the GICA was 2.5 μg/L, and this method can be used for a semi-quantitative determination of clothianidin at three concentration intervals: <2.5 μg/L (negative, −), 2.5−10 μg/L (weakly positive, ±), and >10 μg/L (strongly positive, +). Compared to a previous report on the GICA for clothianidin detection with a visual LOD of 8 μg/L [35], we developed a more sensitive and specific GICA for the rapid test of clothianidin. In addition, multiple batches of the GICA based on different batches of the 1F7-RAb had the same sensitivity (Figure 4C), indicating the full-length IgG RAb-based GICA can be repeated and is suitable for standard production and commercialization.

#### 3.5.2. Matrix Effects

The matrix in samples will affect the specific binding of antigens and antibodies, thus affecting the stability, accuracy, and sensitivity of the method. In this study, a gradient concentration of clothianidin solutions was prepared using diluted samples and compared with strips running with the standard in PBS buffer. The results (Figures S6 and S7) showed that when the agricultural samples (tomatoes and oranges) were diluted six times, and environmental samples (soil and river water) were diluted four times and two times, the impact of the sample matrix on the accuracy and sensitivity could be eliminated.

#### 3.5.3. Application of the GICA in Spiked Samples

The highly specific 1F7-RAb-based GICA was employed in detecting clothianidin in spiked environmental and agricultural samples (Table 2). It was clear that the visual LOD of clothianidin was about 5 ng/mL in river water, 10 ng/g in soil, 15 ng/g in tomatoes, and 15 ng/g in oranges, meeting the requirement of the EU MRL levels (40 ng/g in tomatoes, 60 ng/g in oranges). The results were consistent with the theoretical results, meaning that the highly specific RAb-based GICA has good accuracy and precision, and can be applied to a semi-quantitative determination of clothianidin in environmental and food samples. The results revealed that full-length IgG RAbs could be user-friendly to the development of the stable-performance GICA and further the standardization of downstream immunotest products.

## 4. Conclusions

In this work, using the haptens of clothianidin analogues to prepare competitive antigens for heterologous *ic*-*ELISAs*, we quickly screened out two hybridomas (1F7 and 5C3) secreting anti-clothianidin antibodies. Based on the antibody VR genes, 1F7-RAb and 5C3-RAb in the form of full-length IgG were produced by the mammalian cell HEK293 (F). Using heterologous *ic*-*ELISAs* and non-competitive SPR tests, the 1F7-RAb was verified to be the most specific anti-clothianidin antibody among all the reported anti-clothianidin antibodies, whereas the 5C3-RAb exhibited dual-spectrum recognition towards clothianidin and dinotefuran. Further, the 1F7-RAb was used to develop a portable GICA for the detection of clothianidin residues in environmental and agricultural samples with high sensitivity, specificity, and good reproducibility. To the best of our knowledge, this is the first report to develop a highly specific full-length IgG RAb against clothianidin, as well as its application in an immunoassay. Our work also indicated that the developed full-length RAbs produced by a mammalian cells expression system could be a potential option in addition to traditional antibodies used for the immunoassay of pesticides residues.

## Figures and Tables

**Table 1:** Comparison of the cross-reactivity of anti-clothianidin antibodies with other previously reported antibodies by *ic-ELISAs*.

Neonicotinoid Analogues	PcAb [22]	McAb [23]	McAb [35]	5C3-McAb in This Study	5C3-RAb in This Study	1F7-McAb in This Study	1F7-RAb in This Study
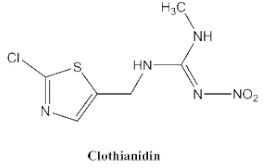	100 ^a^ (46) ^b^	100 (4.4)	100 (25.6)	100 (13.16)	100 (5.22)	100 (5.38)	100 (4.62)
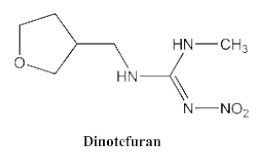	**11.8 ^c^**	**64**	**47.8**	**42.4**	**56**	<0.1	<0.1
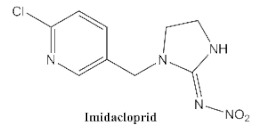	0.8	<0.1	<0.03	<0.1	<0.1	<0.1	<0.1
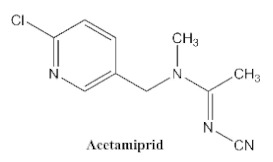	<0.05	<0.1	<0.03	<0.1	<0.1	<0.1	<0.1
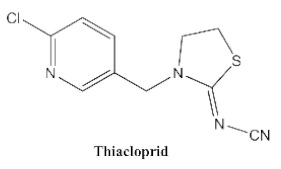	<0.05	<0.1	<0.03	<0.1	<0.1	<0.1	<0.1
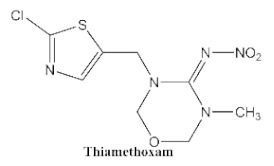	<0.05	<0.1	<0.03	<0.1	<0.1	<0.1	<0.1
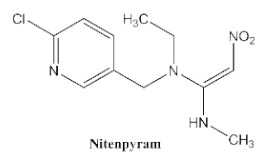	<0.05	<0.1	<0.03	<0.1	<0.1	<0.1	<0.1
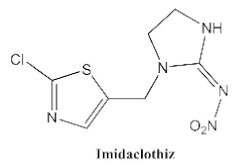	<0.05	<0.1	<0.03	<0.1	<0.1	<0.1	<0.1

^a^ The cross-reactivity rate (%) = Y/Z × 100%, Y: IC_50_ value of clothianidin, Z: IC_50_ value of analogue. ^b^ The numbers in brackets denote IC_50_ value (μg/L). ^c^ Bold figures denote insecticides that showed significant cross-reactivity.

**Table 2:** Detection of clothianidin in the spiked samples GICA. Visual assessment of the test lines was based on three concentration intervals: negative (−), weakly positive (±), and strongly positive (+).

Sample	Spiked Concentration (μg/L or ng/g)	Dilution Time ^d^	Results (*n* = 3)	
			Batch 1 ^e^	Batch 2
River water	0	2	−/−/−	−/−/−
	5		±/±/±	±/±/±
	10		+/+/+	+/+/+
	20		+/+/+	+/+/+
Soil	0	4	−/−/−	−/−/−
	10		±/±/±	±/±/±
	20		+/+/+	+/+/+
	40		+/+/+	+/+/+
Tomato	0	6	−/−/−	−/−/−
	15		±/±/±	±/±/±
	30		+/+/+	+/+/+
	60		+/+/+	+/+/+
Orange	0	6	−/−/−	−/−/−
	15		±/±/±	±/±/±
	30		+/+/+	+/+/+
	60		+/+/+	+/+/+

^d^ The impact of sample matrix on the accuracy and sensitivity could be basically eliminated when samples were diluted at certain times. ^e^ The testing results of GICA based on different batches 1F7-RAb.

## Data Availability

Not applicable.

## Supplementary Materials

The following supporting information can be downloaded at: https://www.mdpi.com/article/10.3390/bios12040233/s1, Figure S1: Chemical structures of haptens for preparation of competitive antigens. (A) the hapten of clothianidin; (B) the hapten of imidaclothiz; (C) the hapten of thiacloprid, Figure S2: Cloning 1F7-McAb and 5C3-McAb to full-length RAbs. (A) The variable region fragments of B2-McAb obtained by 5′RACE PCR. (B) The variable region fragments of G4-McAb obtained by 5′RACE PCR, Figure S3: Cloning 1F7-McAb and 5C3-McAb to full-length RAbs. (A) The variable region fragments of B2-McAb obtained by 5′RACE PCR. (B) The variable region fragments of G4-McAb obtained by 5′RACE PCR, Figure S4: SDS-page of 1F7-RAb (A) and 5C3-RAb (B), Figure S5: McAbs with analytes measured by SPR. (A) Selectivity of antibodies to eight neonicotinoids tested by SPR at the same concentration of 50 nM. (B) 1F7-McAb with clothianidin; (C) 5C3-McAb with clothianidin; (D) 5C3-McAb with dinotefuran; ka: association rate; kd: dissociation rate; KD: dissociation equilibrium constant, Figure S6: Evaluation of matrix effect of GICA on different environmental samples (each group of clothianidin concentrations from left and right to 0, 2.5, 5, 10, 20, 40 μg/L), Figure S7: Evaluation of matrix effect of GICA on different fruit samples (each group of clothianidin concentrations from left and right to 0, 0.063, 1.25, 2.5, 5, 10, 20, 40 μg/L).
