# Supplementary material for: A Novel Full-length IgG Recombinant Antibody Highly Specific to Clothianidin and Its Application in Immunochromatographic Assay
Source: Biosensors (Basel). 2022 Apr 11;12(4):233. doi: 10.3390/bios12040233 (PMC9032790; doi:10.3390/bios12040233)
Supplement: Supplementary file 1 [file biosensors-12-00233-s001.zip › biosensors-1665843-supplementary.pdf]

Article

A Novel Full-Length IgG Recombinant Antibody Highly Specific to Clothianidin and Its Application in Immunochromatographic Assay

Yunyun Chang [1], Yang Chen [1], Shasha Jiao [1], Xinying Lu [1], Yihua Fang [1], Yihua Liu [1,2,*], Ying Zhao [1], Xiuping Zhan [3], Guonian Zhu [1] and Yirong Guo [1,*]

[1] Institute of Pesticide and Environmental Toxicology, Key Laboratory of Biology of Crop Pathogens and Insects of Zhejiang Province, Ministry of Agriculture Key Laboratory of Molecular Biology of Crop Pathogens and Insects, Zhejiang University, Hangzhou 310058, China; 21716193@zju.edu.cn (Y.C.); chengyang96@zju.edu.cn (Y.C.); 21616183@zju.edu.cn (S.J.); 12016122@zju.edu.cn (X.L.); 0915059@zju.edu.cn (Y.F.); zhaoying88@zju.edu.cn (Y.Z.); zhugn@zju.edu.cn (G.Z.)

[2] Research Institute of Subtropical Forestry, Chinese Academy of Forestry, Hangzhou 311400, China

[3] Shanghai Agricultural Technology Extension Service Center, Shanghai 201103, China; zxp19830124@aliyun.com

* Correspondence: liuyh@caf.ac.cn (Y.L.); yirongguo@zju.edu.cn (Y.G.)

1. Experimental Supporting Material

1.1. ELISA procedure

The 96-well plates were coated with competitive antigens at 37°C for 2 h. After washing three times with 0.01 M PBS, the plate was blocked with 2% skimmed milk-PBS, and then washed once. After that, for indirect *ELISA*, 100 μL of antibodies were e put into each well. For indirect competitive *ELISA* (*ic-ELISA*), 50 μL of pesticide solutions and 50 μL of antibodies were put into each well. The plate was washed three times after 1 h incubation at 37 °C. Horseradish peroxidase-goat anti-mouse antibodies were added (100 μL/well) into the plate and incubated for 1 h at 37 °C. After four times' washing, 3,3',5,5'-tetramethylbenzidine was added (100 μL/well) subsequently. After incubation for 15 min at 37°C, the reaction was stopped with 2 mol/L H_2SO_4 (50 μL/well).

As for homologous *ic-ELISA*, CHO-H-OVA was chosen for coating antigen, while IMI-H-OVA, and THC-H-OVA were chosen for heterologous *ic-ELISA*.

1.2. Non-competitive SPR

For selectivity evaluation, eight neonicotinoid pesticides at 100 nM in PBS running buffer were injected over the flow cells at 25 °C with a flow rate of 30 μL/min. The 180 s' association for each sample followed by dissociation for 600 s. Regeneration was conducted to sufficiently remove analyte without reducing antibody binding ability using 1 mM NaOH for 100 s after the dissociation in all further assays.

For kinetics and affinity assay, a gradient dilution series of clothianidin was performed with direct injection for 180 s at a flow rate of 30 μL/ min, followed by a dissociation for 600 s. In order to eliminate the effect of the non-specific binding, the final signals were obtained by subtracting the signal of reference flow cell and blank control. The data were fitted using 1:1 binding kinetic model by Biacore Evaluation Software 3.0 (GE Healthcare, USA). Finally, the dissociation equilibrium constant (KD), dissociation rate (kd) and association rate (ka) were obtained.

1.3. Development of full-length RAb-based GICA for clothianidin detection

Gold nanoparticles-labeled immunoconjugates was developed as follows: firstly, a certain amount of 1F7-RAb was added rapidly into 10 mL of colloidal gold solution with quick stirring for blending. After incubation for 1 h, colloidal gold-labeled immunoconjugate solution was added with the PBS (0.01M, pH7) containing 10% BSA and 1% PEG 20000 (the final concentrations were 1% and 0.1%, respectively) and then incubation for

another 1 h. The solution was centrifuged at 13500 g at 4 °C for 30 min, then the supernatant was removed carefully. The gold-labeled immunoconjugate precipitation was resuspended with washing buffer (1% BSA and 0.1% PEG20000, pH 7). Subsequently, the centrifugation at above-mentioned conditions was carried out again to completely remove the unconjugated antibody. Finally, the gold-labeled immunoconjugate sediment was resuspended with 1 mL of storage buffer (5% sucrose and 1% BSA，pH 7).

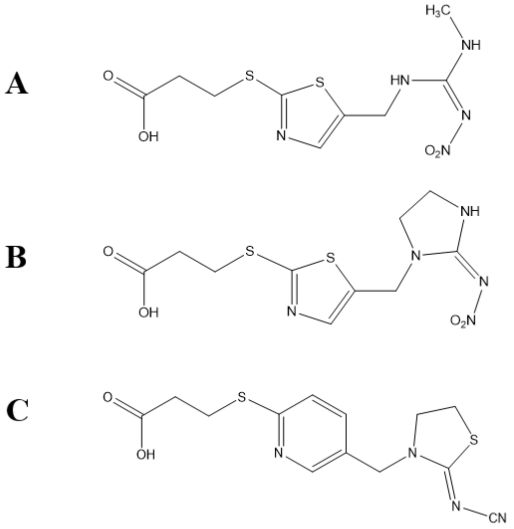

Figure S1. Chemical structures of haptens for preparation of competitive antigens. (A) the hapten of clothianidin; (B) the hapten of imidaclothiz; (C) the hapten of thiacloprid.

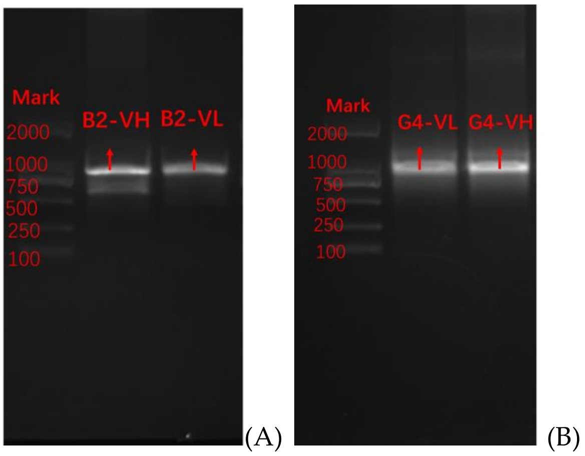

Figure S2. Cloning 1F7-McAb and 5C3-McAb to Full-length RAbs. (A) The variable region fragments of B2-McAb obtained by 5′RACE PCR. (B) The variable region fragments of G4-McAb obtained by 5′RACE PCR.

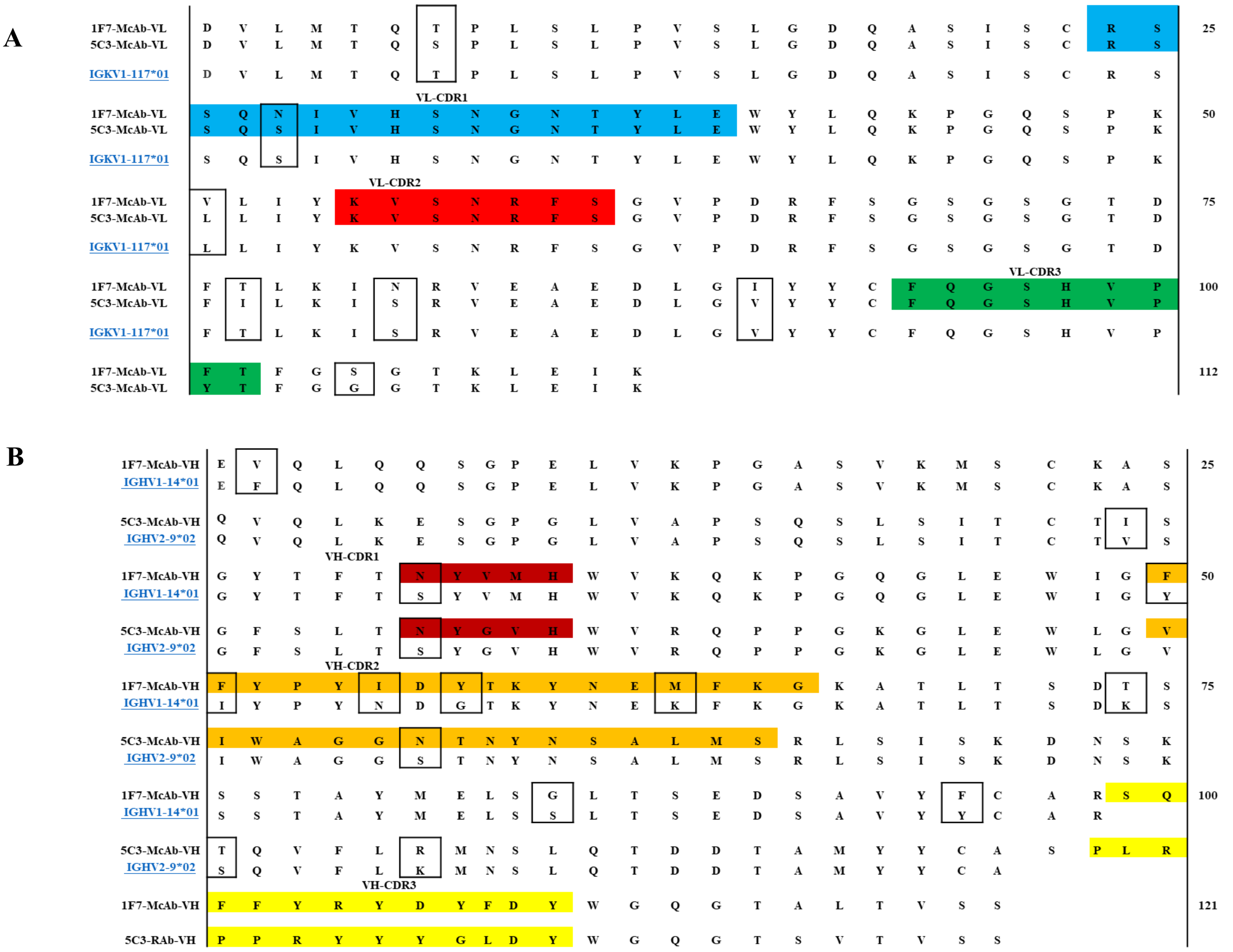

Figure S3. Sequence somatic hypermutation analysis of VLs (A) and VHs (B) by comparison with their germline sequences. The Variable fragments of 5C3-RAb and 1F7-RAb are shown and colored in blue (L-CDR1), red (L-CDR2), green (L-CDR3), dark red (H-CDR1), orange (H-CDR2) and yellow (H-CDR3). The amino acids in the black frames were mutants by comparison with their germline sequences.

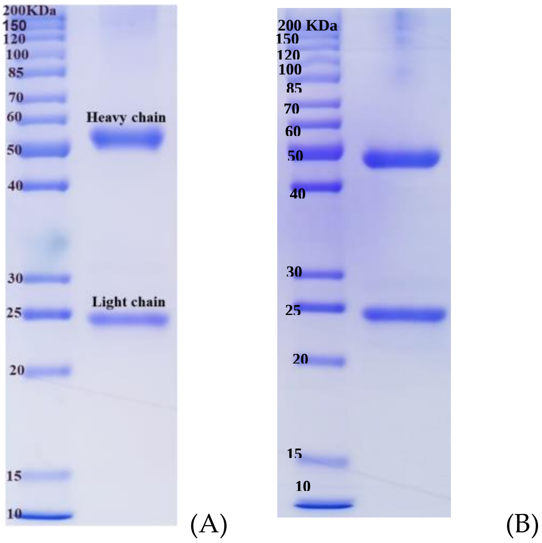

Figure S4. SDS-page of 1F7-RAb (A) and 5C3-RAb (B).

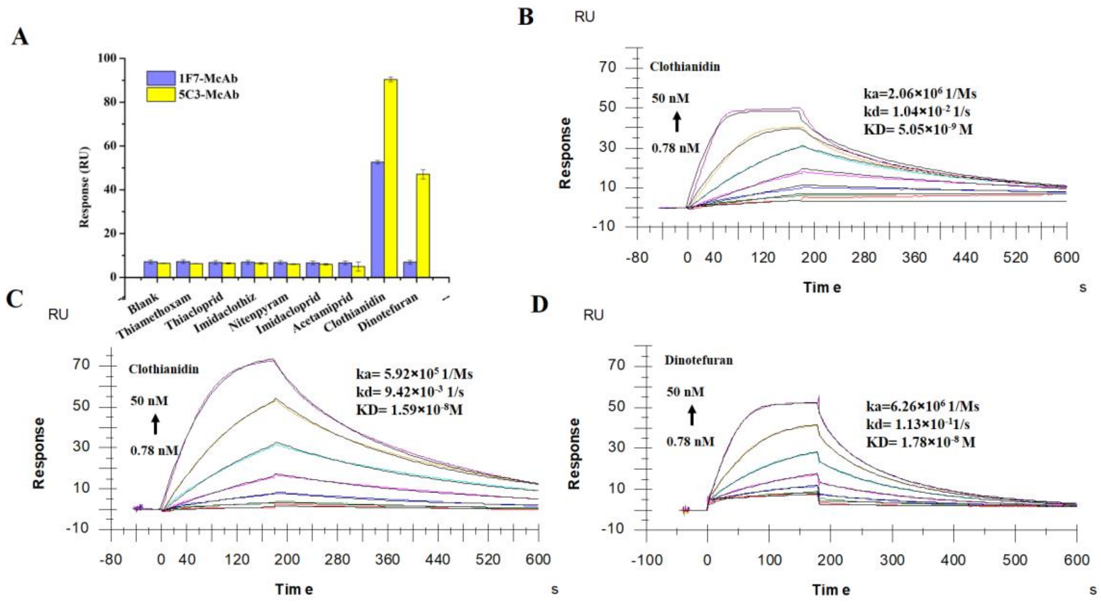

Figure S5. McAbs with analytes measured by SPR. (A) Selectivity of antibodies to eight neonicotinoids tested by SPR at the same concentration of 50 nM. (B) 1F7-McAb with clothianidin; (C) 5C3-McAb with clothianidin; (D) 5C3-McAb with dinotefuran; ka: association rate; kd: dissociation rate; KD: dissociation equilibrium constant.

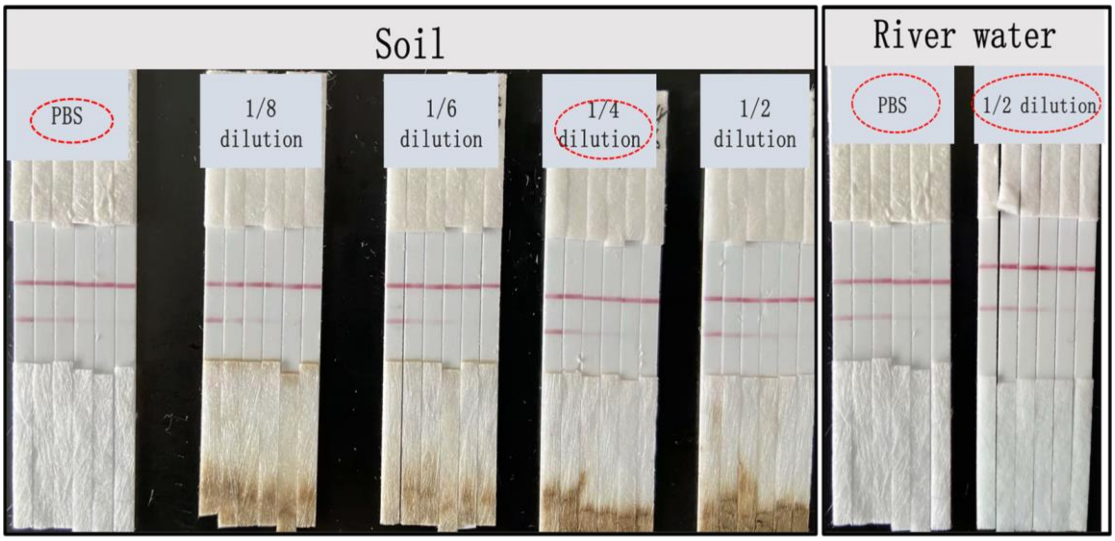

Figure S6. Evaluation of matrix effect of GICA on different environmental samples (each group of clothianidin concentrations from left and right to 0, 2.5, 5, 10, 20, 40 μg/L).

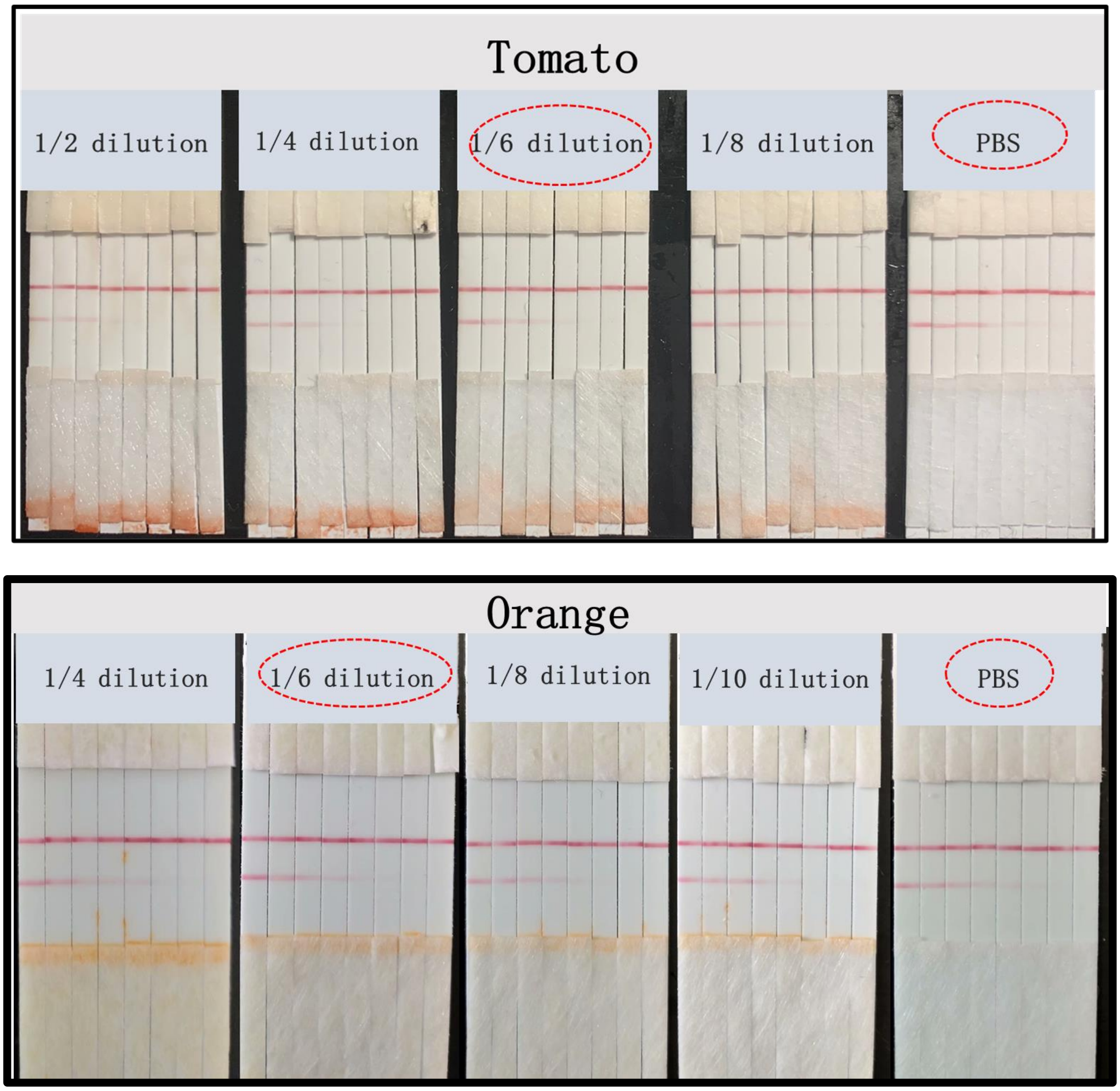

Figure S7. Evaluation of matrix effect of GICA on different fruit samples (each group of clothianidin concentrations from left and right to 0, 0.063, 1.25, 2.5, 5, 10, 20, 40 μg/L).
